# Awareness of the Link Between the Consumption of Ultra-Processed Food and Colorectal Cancer Risk in Saudi Arabia

**DOI:** 10.7759/cureus.33774

**Published:** 2023-01-14

**Authors:** Mohammed Alessa, Maryam O Alarfaj, Hanan A Albenayyan, Almaha A Aleidan, Fatimah A Albahrani, May A Bokhuwah, Raghad M Bukhamsin, Razan M Alzahrani, Mohammed F Alkhalifah, Lamees A Alshekhmobarak, Hajar K Alsaleem, Renad S AlSubaie, Dalal A Almulhim, Aisha A AlJughaiman, Lama A Alobaid

**Affiliations:** 1 General Surgery, King Faisal University, Al-Ahsa, SAU; 2 Medicine, King Faisal University, Al-Ahsa, SAU

**Keywords:** cancer risks, processed food, cross sectional studies, saudi arabia, awareness, colorectal cancer, ultra-processed food

## Abstract

Introduction

Cancer is a group of diseases with uncontrollable growth of abnormal cells. Colorectal cancer (CRC) is one of the most common cancers. Increased intake of animal-source foods, sedentary lifestyle, decreased physical activity, and increased prevalence of excess body weight are independently associated with CRC risk. Additional risk factors include heavy alcohol consumption, cigarette smoking, and consumption of red or processed meat. Ultra-processed food (UPF) is made utilizing multiple components and a number of procedures. Soft drinks and salty or sugary snacks typically contain a lot of added sugar, fats, and processed carbohydrates, which negatively affect the balance of the gut bacteria, nutrients, and bioactive substances that are necessary for the prevention of CRC. The aim of this study is to assess the awareness of the general population in Saudi Arabia toward the relationship between UPF and CRC.

Methods

A cross-sectional questionnaire-based study was conducted in Saudi Arabia between June and December, 2022.

Result

The study involved 802 participants, of which 84% consumed UPF and 71% were aware of the link between UPF and CRC. Only 18.3% were familiar with the particular type of UPF and only 29.4% knew how to prepare them. The prevalence of participants who were aware of the link between UPF and CRC was significantly more among the older age groups, people living in the Eastern Region, and those who knew how to manufacture UPF, while the prevalence of awareness was significantly less among those who regularly consumed UPF.

Conclusion

The study showed that a significant portion of subjects regularly ate UPF, and only a few were aware of its link to CRC. This highlights the need for greater awareness of the fundamentals of UPF and its impact on health. Governmental organizations should develop a strategy to raise public awareness of excessive UPF use.

## Introduction

Cancer is a group of diseases with uncontrollable growth of abnormal cells that invade adjacent structures and spread to other organs. It is the second leading cause of death globally, accounting for an estimated 9.6 million deaths, or one in six deaths. The cancer burden continues to grow globally, exerting tremendous physical, emotional, and financial strain on individuals, families, communities, and health systems [1]. Colorectal cancer (CRC) includes cancer involving the colon, rectum, and anus collectively. Worldwide, CRC is the third most common cancer with more than 1.9 million new cases diagnosed in 2020 [2]. In Saudi Arabia, CRC accounted for 14.6% of total cancers in 2018 [3]. It is estimated to be the second most common cancer following breast cancer in the kingdom. CRC is the most common cancer in Saudi males with 10.6 cases per 100,000 population, whereas it is the third most common cancer among Saudi females with 8.2 cases per 100,000 [3,4]. More than 935,000 CRC deaths were reported globally in 2020 [2]. The World Health Organization (WHO) estimated mortality rates in 2016 to be 10 per 100,000 among Saudi males and seven per 100,000 among females [5].

Unfortunately, CRC in Saudi Arabia is usually diagnosed at advanced stages with metastases. The risk factors for CRC may be genetic, environmental, age-related, gender-related, and other inflammatory conditions of the digestive tract [4]. *KRAS/BRAF* gene mutations have been associated with poor prognosis and short survival [6,7]. Preventive measures include a healthier lifestyle including a balanced diet, not smoking, increased physical activity, and regular screening programs that aim to detect and remove precancerous polyps [2]. Incidence rates have been declining in Western countries due to systematic screening programs while the rates have been increasing considerably in Gulf Cooperation Council (GCC) countries. This is probably due to the increasing prevalence of CRC risk factors such as lack of physical activities, smoking, and unhealthy diet, along with the lack of organized screening programs [7]. Increased intake of animal-source foods, sedentary lifestyle, decreased physical activity, and increased prevalence of excess body weight are independently associated with CRC risk. Additional risk factors include heavy alcohol consumption, cigarette smoking, and consumption of red or processed meat, whereas calcium supplements and adequate consumption of whole grains, fiber, and dairy products appear to decrease risk [2].

Ultra-processed food (UPF) is made utilizing multiple components and a number of procedures. Soft drinks and salty or sugary snacks, reconstituted meat products, and pre-made frozen dishes are all examples of UPF [8]. These foods typically contain a lot of added sugar, fats, and processed carbohydrates, which negatively affect the balance of the gut bacteria, nutrients, and bioactive substances that are necessary for the prevention of colorectal cancer, such as fiber, calcium, and vitamin D, and these are typically lacking in diets rich in UPFs [9]. In addition to having poor nutritional profiles, ultra-processed meals frequently contain food additives such as dietary emulsifiers and artificial sweeteners, some of which have been linked to colon cancer risk by boosting the gut microbiome's pro-inflammatory potential. Additionally, it is possible for probable carcinogens to arise during the cooking of meats containing sodium nitrates, during the heating process, or even when ultra-processed meals are packaged [9]. The rate of consumption of UPF increased dramatically in the previous decades in the United States; the average daily energy intake in the United States in 2009-2010 was 2,069.9 kcal, with 57.5% of calories coming from UPFs. Brazil's population has been observed to consume excessive amounts of UPFs, which result in diets that have high levels of free sugars, total fats, saturated fats, transfats, and low levels of protein [8].

UPF has many health concerns. They come packaged in synthetic materials, which may also have unfavorable impacts on health, and they contain a variety of ingredients that have the potential to seriously harm consumers' health [10]. Over the past few decades, consumption of UPF has increased significantly on a global scale and this expansion is being accompanied by an increase in the prevalence of non-communicable diseases such as type 2 diabetes, hypertension, and cardiovascular diseases; the possibility of getting gestational diabetes and colon cancer is somewhat increased [10]. Due to the carcinogenic qualities (such as acrylamide, heterocyclic amines, and polycyclic aromatic hydrocarbons) that are found in UPF products as a result of the Maillard reaction, it can raise the risk of colon cancer. Also, compounds suspected to have carcinogenic and endocrine-disrupting qualities may be present in the packaging of UPFs, such as bisphenol A [11].

According to the American Cancer Society (ACS), one of the risk factors for CRC is the high consumption of red and processed meats, and low dietary fiber [12]. This is due to the fact that obesogenic properties and exposure to potentially carcinogenic compounds such as certain food additives and neoformed processing contaminants lead to increase cancer risk [13]. Up to 10% of UPF intake was associated with higher overall cancer risk [14]. The potential role of food additives in altering the gut microbiota, promoting inflammation, and contaminants formed during food processing or migrated from food packaging may all promote cancer development [15]. Males in the highest 20th percentile for consumption of UPF had a 29% increased risk of colorectal cancer, especially distal colon cancer [16]. Alkhayyat et al. did a cross-sectional study conducted in Saudi Arabia for 4090 males and females to assess public awareness about CRC. This study suggests that females are more aware that red or processed meat is a risk factor for CRC. In addition, older participants knew that eating red or processed meat increases the risk of developing CRC compared to younger adults [17]. Azzeh et al. did a case-control study for 137 patients with colon and/or rectal cancer in the Mecca region of Saudi Arabia to investigate the risks and protective benefits of dietary factors affecting CRC [18]. This study suggested that there was no effect of processed meats on the incidence of CRC. Deoula et al. did a case-control study to examine the association between CRC risk and consumption of meat, considering different subgroups, such as red meat, white meat, traditionally processed meat, and industrially processed in the Moroccan population [19]. This study suggested that higher consumption of poultry and red meat increases the risk of colon cancer and CRC, but not of rectum cancer. In addition, the consumption of industrially processed meat increased the risk of all CRC sites. However, there was an inverse relationship between traditionally processed meat and colon cancer, and overall CRC risk. In a review by Tabung et al. of the literature published between 2000 and 2017, searching for an association between dietary patterns and CRC risk, it was suggested that a higher intake of processed meat is associated with a higher risk of CRC [20].

Mehta et al. did Cox proportional hazards models for up to 32 years of follow-up of 137,217 participants to examine associations between Western and prudent dietary patterns and CRC risk in the Health Professionals Follow-up Study and Nurses’ Health Study [21]. The result suggests that Western dietary patterns that are rich in red and processed meats, high-fat dairy, refined grains, and desserts are at high risk of distal colon and rectal tumors. However, prudent dietary patterns characterized by high intakes of vegetables, fruits, whole grains, and fish are at low risk of CRC. A study by Wang et al. examined the relationship between the intake of UPFs and the risk of CRC using three large prospective cohorts in the United States [22]. The study suggested that high consumption of total UPFs in males and certain subgroups of UPFs in males and females were highly associated with an increased risk of CRC. In addition, the association between UPF consumption and CRC among males was independent of BMI.

Romaguera et al. did a metacentric population-based case-control study in Spain to study whether the consumption of UPFs and drinks is associated with CRC and breast and prostate cancers. The study suggested that there was an association between the consumption of UPFs and drinks and CRC. However, there was no association between the consumption of UPFs and drinks and breast and prostate cancers [23]. A population-based cohort study was done by Fiolet et al, to assess the prospective associations between the consumption of UPF and the risk of cancer [24], and suggested that there is a positive association between industrially processed meat and the risk of CRC, colon cancer, and rectal cancer.

The aim of the current study is to assess the awareness of the general population in Saudi Arabia regarding the relationship between UPF and CRC.

## Materials and methods

This was a community-based cross-sectional study conducted in Saudi Arabia between June 2022 and December 2022. Prior to conducting the survey, ethical approval was obtained from the Institutional Research Board and the Ethics Committee of King Faisal University in Al-Ahsa city, Saudi Arabia (Approval number: KFU-REC-2022-DEC-ETHICS397).

Study size and population

Any Saudi resident above the age of 12 years is included in the selection criteria. Non-Saudi residents, non-completed questionnaires, and individuals younger than 12 years old were excluded from the study. According to the General Authority of Statistics in Saudi Arabia, the total Saudi population is 35 million approximately in 2021. Therefore, a sample of at least 382 Saudi residents was required to participate in our study as calculated by the following statistical formula based on a study population: confidence interval is 95%; margin of error is $5\% n = P (1-P) (Za/2) 2 (E) 2$, where n = population size, Za= critical value of the normal distribution at the required confidence level, P = sample proportion, E = margin of error. We ultimately included 802 participants in our study. 

Statistical analysis

The data analyses for this project were performed using IBM SPSS Statistics for Windows, Version 26.0 (Released 2019; IBM Corp., Armonk, New York, United States). Descriptive statistics were presented using numbers and percentages. The relationship between the awareness of the UPF-CRC link and the socio-demographic characteristics and the knowledge about UPF was analyzed using the Chi-square test. Identified significant results in the bivariate analysis were gathered into a multivariate regression model to determine the independent significant factor of being aware of the link between UPF and CRC with corresponding odds ratio as well as 95% confidence interval. P-value <0.05 has been accepted as the significant level for all statistical tests.

Data collection

A survey questionnaire was distributed to collect data regarding sociodemographics, knowledge of processed food, and the rate of consumption (Table 1). The survey was conducted in Arabic, the primary language of the targeted population. The predesigned questionnaire was distributed among the community through Whatsapp (Whatsapp LLC, Menlo Park, California, United States). Consent from the participants was taken prior to their filling out the form.

**Table 1:** English translation of the survey questionnaire

Knowledge about the association between ultra-processed food and colorectal cancer
Carbonated soft drinks, sweetened juices, and dairy drinks, powders for juices, and 'energy drinks. Sugar substitutes, sweeteners, and all syrups sausages, chicken and fish nuggets or sticks, and other pre-prepared frozen dishes. dried products such as cake mix, powdered soup, and instant noodles. packaged snacks, morning cereals, and cereal bars. Do you know what type of food these are considered to be?
Unprocessed or minimally processed food
Processed culinary ingredients
Processed food
Ultra-processed food
Do you consume these types of food?
No
Yes
If yes, it forms:
10-20% of your diet
21-50% of your diet
More than 50% of your diet
After knowing that ultra-processed food increases the risk of cancer, do you think that you would decrease the rate of consumption of this type of food?
No
Yes

## Results

This cross-sectional survey involved 802 participants. Table 2 presents the basic demographic characteristics of participants. The most common age group was 20-39 years (42.1%) with females being dominant (78.1%). Respondents who live in the Eastern Region constitute 52.7%. With regard to education, the majority had bachelor's or higher degrees (66.5%).

**Table 2:** Basic demographic characteristics of participants (n=802)

Study data	N (%)
Age group	
12-19 years	109 (13.6%)
20-39 years	338 (42.1%)
40-59 years	291 (36.3%)
≥60 years	64 (08.0%)
Gender	
Male	176 (21.9%)
Female	626 (78.1%)
Region of residence	
Eastern Region	423 (52.7%)
Central Region	168 (20.9%)
Western Region	87 (10.8%)
Northern Region	57 (07.1%)
Southern Region	67 (08.4%)
Educational level	
Secondary or below	175 (21.8%)
Diploma holder	94 (11.7%)
Bachelor or higher	533 (66.5%)

The assessment of the knowledge about the association between UPF and CRC is given in Table 3. It can be observed that 18.3% were aware of the various types of UPF and 29.4% knew how to cook them. The prevalence of participants who consumed UPF was 84.2%. Approximately 35.2% regularly ate UPF, which comprised 10-20% of their daily diet. The prevalence of participants who were aware of the link between UPF and CRC was 71.4%.

**Table 3:** Assessment of knowledge about the association between ultra-processed food and colorectal cancer (n=802) UPF: ultra-processed food; CRC: colorectal cancer

Statement	N (%)
Soft drinks, sweetened juices, dairy drinks, juice powders, energy drinks, natural sugar substitutes, sausages, pepperoni, mortadella, frozen chicken and fish, and other pre-prepared frozen dishes. Do you know what kind of these foods are?	
Unprocessed or minimally	54 (06.7%)
Processed culinary ingredients	30 (03.7%)
Processed food	571 (71.2%)
Ultra-processed food	147 (18.3%)
Do you know how to manufacture this type of food?	
Yes	236 (29.4%)
No	566 (70.6%)
Do you consume these types of food?	
Yes	675 (84.2%)
No	127 (15.8%)
How much do you consume in your daily diet?	
<10% of daily diet	14 (01.7%)
10-20% of daily diet	282 (35.2%)
21-50% of daily diet	257 (32.0%)
>50% of daily diet	122 (15.2%)
I do not consume this type of food	127 (15.8%)
Are you aware of the link between UPF and CRC?	
Yes	573 (71.4%)
No	229 (28.6%)

When measuring the relationship between the awareness of UPF and CRC-linked in terms of the sociodemographic characteristics and the knowledge of UPF (Table 4), it was found that the prevalence of participants who were aware of the UPF and CRC link was significantly more common among the older age group (p<0.001), those living in the Eastern Region (p<0.001), and those who knew how to manufacture UPFs (p=0.034), while the prevalence of awareness was significantly less among those who regularly consumed UPFs (p<0.001).

**Table 4:** Relationship of the awareness of the UPF and CRC link with sociodemographic characteristics and knowledge about UPF (n=802) UPF: ultra-processed food; CRC: colorectal cancer § P-value has been calculated using the Chi-square test; ** Significant at p<0.05 level

Factor	Awareness of UPF and CRC link		P-value ^§^
	Aware N (%) ^(n=573)^	Not Aware N (%) ^(n=229)^	
Age group			
<40 years	275 (48.0%)	172 (75.1%)	<0.001 **
≥40 years	298 (52.0%)	57 (24.9%)	
Gender			
Male	133 (23.2%)	43 (18.8%)	0.171
Female	440 (76.8%)	186 (81.2%)	
Region of residence			
Inside Eastern Region	326 (56.9%)	97 (42.4%)	<0.001 **
Outside Eastern Region	247 (43.1%)	132 (57.6%)	
Educational level			
Diploma or below	195 (34.0%)	74 (32.3%)	0.642
Bachelor or higher	378 (66.0%)	155 (67.7%)	
Knowledge about UPF			
Yes	112 (19.5%)	35 (15.3%)	0.159
No	461 (80.5%)	194 (84.7%)	
Knowledge of manufacturing UPF			
Yes	181 (31.6%)	55 (24.0%)	0.034 **
No	392 (68.4%)	174 (76.0%)	
Regular consumption of UPF			
Yes	458 (79.9%)	217 (94.8%)	<0.001 **
No	115 (20.1%)	12 (05.2%)	

When conducting multivariate regression estimates to determine the independent significant factor associated with knowing the UPF and CRC link (Table 5), it was observed that the older age group and knowledge of manufacturing UPF were the independent significant predictors of increased awareness about the UPF and CRC link while regular consumption of UPF was the independent significant factor of decreased awareness of the UPF and CRC link. This further indicates that compared to the younger age group (<40 years), the odds of being aware of the UPF and CRC link among older participants (≥40 years) were predicted to increase by at least 2.64 times higher (adjusted odds ratio (AOR)=2.641; 95%CI=1.798-3.879; p<0.001). Participants who knew how to cook UPF were 1.48 times more likely to be aware of the UPF and CRC link compared to those who did know how to cook UPF (AOR=1.483; 95%CI=1.029-2.137; p=0.035). In contrast, participants who were eating UPF regularly were significantly less likely to be aware of the UPF and CRC link (AOR=0.342; 95%CI=0.181-0.648; p=0.001).

**Table 5:** Multivariate regression analysis to determine the independent significant factor associated with knowing the UPF and CRC link (n=802) UPF: ultra-processed food; CRC: colorectal cancer; AOR: adjusted odds ratio § P-value has been calculated using the Chi-square test; ** Significant at p<0.05 level.

Factor	AOR	95% CI	P-value
Age group			
<40 years	Ref		
≥40 years	2.641	1.798 – 3.879	<0.001 **
Region of residence			
Inside Eastern Region	Ref		
Outside Eastern Region	0.872	0.615 – 1.237	0.444
Knowledge of manufacturing UPF			
Yes	1.483	1.029 – 2.137	0.035 **
No	Ref		
Regular consumption of UPF			
Yes	0.342	0.181 – 0.648	0.001 **
No	Ref		

## Discussion

The present study was carried out to determine the awareness of the general population regarding the link between UPF (soft drinks, sweetened juices, dairy drinks, juice powders, energy drinks, natural sugar substitutes, sausages, pepperoni, mortadella, frozen chicken and fish, and other pre-prepared frozen dishes) and CRC. The findings of this study revealed that the prevalence of participants who were aware of the link between UPF and CRC was 71.4%. This prevalence was consistent with a study done in Qatar [25]. Based on the report, approximately 71.7% of the Qatari population was aware that eating processed meat regularly has a positive association with CRC. However, in Egypt, it was found that the awareness of the Egyptian population regarding CRC risk factors was insufficient, with only 42.7% being aware that eating red meat or processed meat was a risk factor for CRC and only 47.9% knew that low fruit and raw vegetable consumption could also be risk factors for CRC [26]. However, in a large prospective study conducted in France, they found out that UPF intake was associated with greater cancer risk [14], while in Morocco, red meat intake was positively associated with colon cancer and CRC risk but no significant association was seen between red meat intakes and rectal cancer risk [19]. In Spain, a study found that regular consumption of UPF and drinks was linked to an increased risk of CRC but no differences with breast cancer or prostate cancer [23]. In contrast, Wang et al. found no difference between UPF intakes and the risk for CRC among females, but they found significant differences in males [9]. Awareness campaigns are necessary to inform the public about the association between UPF and CRC. Healthcare providers should promote awareness of healthy lifestyles and cancer prevention among the public.

Data from this study indicate that the older age group (≥40 years) and the knowledge of how to manufacture UPF were the factors independently associated with increased awareness of the UPF and CRC link. In a study by Al-Dahshan et al., they documented that female participants had better awareness regarding the link between CRC and the consumption of processed meat, eating red meat, and low consumption of fruits and vegetables [25]. In our study, however, there was no indication of any relationship between the awareness of the UPF and CRC link and gender and education; however, we found a significant association in terms of region of residence.

We learned that participants who eat UPF regularly are at decreased odds of being aware of the UPF and CRC link. This is almost consistent with the reports documented by Azzeh et al., wherein dairy product intake of one to five servings per day, legume intake of three to five servings per week, leafy vegetable intake of one to five servings per week, black tea intake of three or more cups a day, coffee intake of one or more cups per day, and olive oil intake of one to five servings per week were found to reduce the risk of CRC [18]. In a study carried out by Lima and Gomes-da-Silva, they revealed that the preventive factors for CRC include increased consumption of a wide variety of fruit and vegetable, particularly, dark-green leafy, deep-yellow tones, cruciferous, and fiber [27]. However, when Huybrechts et al. assessed the effect of UPF on breast cancer, they discovered that industrial bread, packaged sweet and savory snacks, breakfast cereals, cakes and desserts, and ready-eat/fast food intakes were the factors most strongly associated with breast cancer [28]. In contrast, Egeberg et al. found no significant association between intake of red meat, processed meat, fish, or poultry and risk for colon cancer or rectal cancer [29].

Regarding the specific knowledge of the Saudi population about UPF, it was seen in the current study that although a great proportion of the participants regularly consumed UPF (84.2%), comprising about 10-20% of their daily diet (35.2%), only 18.3% were familiar with the specific type of UPF and only 29.4% were aware of how to cook them. This scenario underscores the need for more awareness regarding the basic facts of UPF and its effect on health. Government institutions should devise a plan to increase the awareness of the public toward the excessive consumption of UPF.

Limitations of the study include: the data was self-reported and hence may have introduced some bias and the majority of the participants were from the Eastern Region. The strengths of the study include its large sample size and the fact that there has been no previous study in the same area of research in Saudi Arabia.

## Conclusions

The study showed that a significant portion of the participants regularly ate UPF, and only a few were aware of its link to CRC. This highlights the need for greater awareness of the fundamentals of UPF and its impact on health. Governmental organizations should develop a strategy to raise public awareness of excessive UPF use.
